# Distinct mechanisms of spike timing‐dependent LTD at vertical and horizontal inputs onto L2/3 pyramidal neurons in mouse barrel cortex

**DOI:** 10.1002/phy2.271

**Published:** 2014-03-26

**Authors:** Abhishek Banerjee, Ana González‐Rueda, Cassandra Sampaio‐Baptista, Ole Paulsen, Antonio Rodríguez‐Moreno

**Affiliations:** 1 Department of Physiology, Anatomy and Genetics, The Neuronal Oscillations Group, Oxford, UK; 2 Department of Physiology, Development and Neuroscience, University of Cambridge, Cambridge, UK; 3 Department of Physiology, Anatomy and Cellular Biology, University Pablo de Olavide, Seville, Spain; Abhishek Banerjee, The Picower Institute for Learning and Memory, Department of Brain and Cognitive Sciences, Massachusetts Institute of Technology, Cambridge, 02139, MA

**Keywords:** LTD, LTP, mouse, somatosensory cortex, STDP

## Abstract

Spike timing‐dependent plasticity (STDP) is an attractive candidate to mediate the synaptic changes that support circuit plasticity in sensory cortices during development. STDP is prevalent at excitatory synapses, but it is not known whether the underlying mechanisms are universal, or whether distinct mechanisms underpin STDP at different synapses. Here, we set out to compare and contrast STDP at vertical layer 4 and horizontal layer 2/3 inputs onto postsynaptic layer 2/3 neurons in the mouse barrel cortex. We find that both vertical and horizontal inputs show STDP, but that they display different time windows for induction of timing‐dependent long‐term depression (t‐LTD). Moreover, whereas t‐LTD at vertical inputs requires presynaptic NMDA receptors and is expressed presynaptically, using paired recordings we find that t‐LTD at horizontal inputs requires postsynaptic NMDA receptors and is expressed postsynaptically. These results demonstrate that similar forms of plasticity on the same postsynaptic neuron can be mediated by distinct mechanisms, and suggest that these forms of plasticity may enable these two types of cortical synapses to support different functions.

## Abstract (precis)

Timing‐dependent LTD (t‐LTD) at vertical inputs on layer 2/3 neurons (L4‐L2/3) requires presynaptic NMDA receptors and is expressed presynaptically, but little is known about these mechanisms at horizontal inputs (L2/3‐L2/3). Using paired recordings we demonstrate here that t‐LTD at L2/3‐L2/3 synapses also requires NMDA receptors but is induced and expressed postsynaptically. These results indicate that similar forms of plasticity on the same postsynaptic neuron may be mediated by distinct mechanisms and suggest that these forms of plasticity may support different developmental functions in the cortex.

## Introduction

Synapses in the superficial layers of primary somatosensory cortex (S1) provide a good model system in which to study synaptic mechanisms of receptive field plasticity (Feldman and Brecht 2005; Petersen and Crochet 2013). Alterations in sensory experience modify the response properties of neurons to sensory stimuli. In the mouse barrel cortex, this has been ascribed to potentiation of vertical excitatory inputs onto layer 2/3 neurons (Clem et al. 2008), which, in turn, can alter synaptic drive onto other layer 2/3 neurons as they are highly reciprocally connected (Lefort et al. 2009). Horizontal inputs appear to exhibit a longer critical period of plasticity compared to that of vertical inputs onto layer 2/3 neurons (Wen and Barth 2011).

Input‐specific differences of experience‐dependent changes in the strength of excitatory synapses may be important during development. An attractive mechanism for experience‐dependent synaptic changes is spike timing‐dependent plasticity (STDP; Feldman 2012). Canonical STDP is a Hebbian learning rule (Caporale and Dan 2008), in which the order and precise temporal interval of spiking in presynaptic and postsynaptic neurons determine the direction and magnitude of changes in synaptic weight (Markram et al. 1997; Bi and Poo 1998; Debanne et al. 1998). STDP is found in many brain regions, including rodent visual (Sjöström et al. 2003; Froemke et al. 2005; Corlew et al. 2007) and barrel cortex (Feldman 2000). At barrel cortex layer 4‐to‐layer 2/3 connections, timing‐dependent long‐term potentiation (t‐LTP) requires postsynaptic NMDA receptors and is expressed postsynaptically (Bender et al. 2006; Nevian and Sakmann 2006; Rodriguez‐Moreno and Paulsen 2008; Rodriguez‐Moreno et al. 2011; Itami and Kimura 2012), whereas timing‐dependent long‐term depression (t‐LTD) requires activation of presynaptic NMDA receptors and is expressed presynaptically (Rodriguez‐Moreno and Paulsen 2008).

Although the mechanisms of STDP at the vertical layer 4 input onto layer 2/3 pyramidal neurons have been studied in some detail (Banerjee et al. 2009; Feldman 2012), STDP at the horizontal layer 2/3 input onto layer 2/3 pyramidal neurons in rodent barrel cortex has been less studied, and it is not known whether STDP follows the same rules and mechanisms at horizontal as at vertical connections. Therefore, we set out to compare and contrast STDP at vertical and horizontal inputs onto layer 2/3 pyramidal neurons during the critical period of mouse barrel cortex development. We found that both vertical and horizontal pathways express STDP, but that they exhibit distinct mechanisms of t‐LTD. Although t‐LTD at the vertical input requires presynaptic NMDA receptors and is expressed presynaptically, t‐LTD at the horizontal input requires postsynaptic NMDA receptors and is expressed postsynaptically. These differences suggest that they subserve distinct functional roles during development.

## Methods

### Ethical approval

All animal procedures were in accordance with the UK Animals (Scientific Procedures) Act 1986 and performed under appropriate Home Office personal and project licenses. C57BL/6 mouse pups at postnatal day 12–18 were anesthetized using isoflurane (2%) and decapitated for slice preparation. A total of 70 animals were used in these experiments and slices from two to five mice were used in each type of experiment.

### Thalamocortical slice preparation and whole‐cell recording

Thalamocortical slices from the barrel cortex were prepared (Agmon and Connors 1991; Banerjee et al. 2009) and maintained at room temperature (22–27°C) in artificial cerebrospinal fluid (aCSF) containing (in mmol/L): NaCl 126; KCl 3; NaH_2_PO_4_ 1.25; MgSO_4_ 2, CaCl_2_ 2, NaHCO_3_ 26; glucose 10, pH 7.2–7.4; bubbled with carbogen gas (95% O_2_/5% CO_2_). Whole‐cell recordings were made from layer 2/3 pyramidal neurons with 5–7 MΩ borosilicate pipettes with a pipette solution containing (in mmol/L): potassium gluconate 115; HEPES 40; NaCl 4; ATP‐Mg 2; GTP 0.3, adjusted to pH 7.2 with KOH. Data were recorded using an Axoclamp‐2B or Multiclamp‐700B amplifier (Molecular Devices, Sunnyvale, CA), low‐pass filtered at 1 or 2 kHz and acquired at 5 kHz, using an Instrutech ITC‐16 or ITC‐18 AD board on a PC running Igor Pro (WaveMetrics, Lake Oswego, OR). Only cells with a stable resting membrane potential negative to −50 mV, overshooting action potentials (exceeding 75–80 mV threshold‐to‐peak) and an input resistance >100 MΩ were included. Cells were rejected if series resistance changed by more than 15%. All recordings were made at room temperature (22–24°C).

### Timing‐dependent plasticity protocols

**Figure 1.**
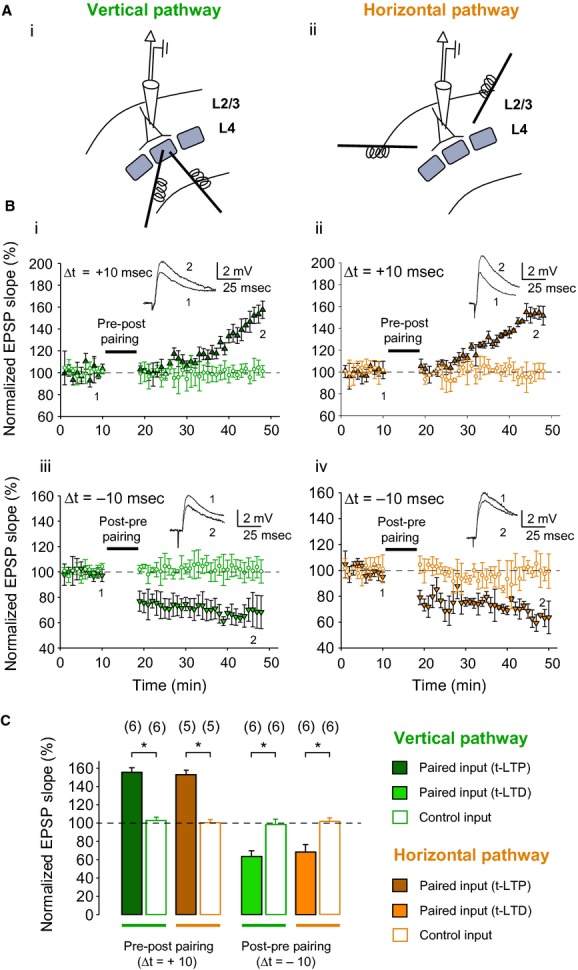
Spike timing‐dependent plasticity at vertical and horizontal inputs on layer 2/3 neurons in mouse barrel cortex. (A) Schematic diagrams of a layer 2/3 pyramidal neuron with patch pipette at the soma, and two extracellular stimulation electrodes at the base of a layer 4 barrel to study vertical inputs (Ai) or at neighboring layer 2/3 barrel column to study horizontal inputs (Aii). (B) STDP at vertical and horizontal inputs. Pre‐before‐post pairing induces t‐LTP at both vertical (Bi) and horizontal inputs (Bii), and post‐before‐pre pairing induces t‐LTD at both vertical (Biii) and horizontal inputs (Biv). Δt is the time interval between EPSP onset and peak of postsynaptic spike. Synaptic efficacy was monitored over time in paired (triangles) and unpaired control pathway (circles). Traces show EPSPs before (1) and 30 min after (2) pairing. (C) Summary of results. Error bars are SEM. *P < 0.05, paired two‐sample *t*‐test. The number of slices used for each protocol is indicated in parentheses at the top of each error bar.

Excitatory postsynaptic potentials (EPSPs) were recorded in whole‐cell, current‐clamp mode from layer 2/3 pyramidal neurons in mouse barrel cortex. Cells were held at approximately −70 mV by a small negative current. EPSPs were elicited alternately in two input pathways by brief current pulses (50 *μ*s; 50–100 *μ*A; test and control pathway, each at 0.2 Hz) applied via two monopolar extracellular stimulation electrodes placed either at the base of the barrel in layer 4 (vertical pathway; Fig. 1Ai) or in neighboring layer 2/3 barrel columns (horizontal pathway; Fig. 1Aii). Stimulus intensity was adjusted until a clear small monosynaptic postsynaptic response (2–5 mV) was evoked in each pathway.

After recording a stable EPSP baseline period of at least 10 min (<10% drift), a plasticity protocol (pre‐before‐post or post‐before‐pre) was applied to the test pathway. Plasticity was induced by pairing synaptic responses with a single postsynaptic action potential evoked by a brief (5–10 msec) current pulse (200 pA) through the postsynaptic patch pipette at 0.2 Hz, repeated 100 times, following which the EPSP was monitored for a further 20–30 min.

### Paired recordings

One recording pipette was placed in layer 2/3 in one barrel column and another within 200–300 *μ*m in layer 2/3 of the same or a neighboring barrel column. Once dual whole‐cell patch‐clamp configuration was achieved, a brief current pulse was repeatedly applied to the presynaptic neuron to test whether presynaptic action potentials evoke EPSPs in the postsynaptic neuron. Only pairs in which the stimulation of one layer 2/3 neuron produced a monosynaptic EPSP in the other layer 2/3 neuron were used for further analysis. The protocol used to induce t‐LTD was otherwise identical to that used during extracellular stimulation.

### MK801 experiments

t‐LTD was induced in current‐clamp mode. After 20 min of assessing the synaptic weights following the plasticity protocol, extracellular stimulations were stopped and 6‐cyano‐7‐nitroquinoxaline‐2,3‐dione (CNQX, 5 *μ*mol/L), gabazine (2 *μ*mol/L) and MK801 (10 *μ*mol/L) were added through the superfusion system. Ten minutes later, NMDA receptor‐mediated EPSCs were evoked alternately in the test and control pathway at 0.2 Hz in voltage‐clamp mode at a holding potential of +60 mV. NMDA‐EPSCs were measured over 220 repetitions. The decay in NMDA‐EPSC peak amplitude with each stimulation was fitted with a single exponential function. The time constant of the decay was measured for each experiment and averaged over all experiments of the same type.

### Data analysis and statistics

Plasticity was assessed measuring the slope of the rising phase of the EPSP as a linear fit between time points corresponding to 25–30% and 70–75% of the peak amplitude during control conditions (Rodriguez‐Moreno and Paulsen 2008; Banerjee et al. 2009). The time window of plasticity was assessed by varying the time interval (Δt) between presynaptic stimuli (or spikes) and postsynaptic spikes, with positive values indicating that the postsynaptic action potential occurred after the presynaptic stimulus and negative values indicating that the postsynaptic action potential occurred before the presynaptic stimulus (or spike). The last 5 min of recording was used to estimate changes in synaptic efficacy compared to baseline. For paired‐pulse ratio (PPR) experiments, two EPSPs were evoked 40 msec apart for 2 min at baseline frequency (24 times) at the beginning of the baseline recording and again 20 min after applying the pairing protocol. The PPR was expressed as the slope of the second EPSP divided by the slope of the first EPSP. Statistical comparisons were made using one‐sample, as well as unpaired and paired two‐sample Student's *t*‐tests as indicated in each case. *P*‐values < 0.05 were considered significant. Data are presented as mean ± SEM unless otherwise indicated. Drugs were purchased from Tocris Bioscience (Dorset, UK) and Sigma (Bicester, UK).

## Results

### Spike timing‐dependent plasticity at vertical and horizontal inputs on layer 2/3 neurons in mouse barrel cortex

First, in order to confirm that both vertical and horizontal inputs onto layer 2/3 pyramidal neurons in mouse barrel cortex show STDP, we made whole‐cell recordings from layer 2/3 pyramidal neurons, while placing two stimulation electrodes either in layer 4 of the same barrel column (vertical pathway; Fig. 1Ai) or in layer 2/3 of a neighboring barrel column (horizontal pathway; Fig. 1Aii).

Both the vertical and horizontal pathways showed STDP with 10 msec positive or negative time interval between presynaptic stimulation and postsynaptic action potential. Consistent with previous reports (Feldman 2000; Rodriguez‐Moreno and Paulsen 2008; Banerjee et al. 2009), significant t‐LTP occurred in the vertical pathway following a pre‐before‐post single‐spike pairing protocol (155 ± 5%; *P* < 0.05, one‐sample *t*‐test, *n* = 6), while an unpaired pathway remained unchanged (103 ± 3%, *P* > 0.05; one‐sample *t*‐test, *n* = 5; Fig. 1Bi). Similarly, in the horizontal pathway, significant t‐LTP was seen following a pre‐before‐post single‐spike pairing protocol (153 ± 5%; *P* < 0.05, one‐sample *t*‐test, *n* = 5), while an unpaired pathway remained unchanged (100 ± 3%, *P* > 0.05; one‐sample *t*‐test, *n* = 5; Fig. 1Bii). Conversely, a post‐before‐pre pairing protocol induced robust t‐LTD in the vertical pathway (63 ± 6%; both *P* < 0.05, one‐sample *t*‐test, *n* = 5), whereas an unpaired pathway remained unchanged (98 ± 6%; p > 0.05, one‐sample *t*‐test, n = 5; Fig. 1Biii); a post‐before‐pre pairing protocol also induced robust t‐LTD in the horizontal pathway (68 ± 8%; *P* < 0.05, one‐sample *t*‐test, *n* = 5), while an unpaired pathway remained unchanged (102 ± 4%, *P* > 0.05; one‐sample *t*‐test, *n* = 5; Fig. 1*Biv*). Thus, input‐specific t‐LTP and t‐LTD could both be induced at both vertical and horizontal inputs onto postsynaptic layer 2/3 neurons in mouse barrel cortex in 12–18 days old mice (Fig. 1C).

### Time window for plasticity induction in layer 2/3 neurons

To compare the time window for induction of plasticity at vertical and horizontal inputs on layer 2/3 pyramidal neurons, we paired presynaptic stimulation and single postsynaptic spikes, and tested a 50 msec time interval for t‐LTP induction as well as three additional time intervals for t‐LTD induction; 50 msec, 100 msec, and 200 msec.

### Time window for t‐LTP induction at vertical and horizontal inputs

In contrast to pre‐before‐post pairing with a 10 msec time difference between presynaptic stimulation and postsynaptic action potential, pre‐before‐post pairing with a longer time interval (Δt = 50 msec) did not elicit t‐LTP in either vertical or horizontal pathways (vertical input, 96 ± 7%; *P* > 0.05, one‐sample *t*‐test, *n* = 6; horizontal input, 94 ± 8%; *P* > 0.05, one‐sample *t*‐test, *n* = 6).

### Time window for t‐LTD induction at the vertical and horizontal inputs

**Figure 2.**
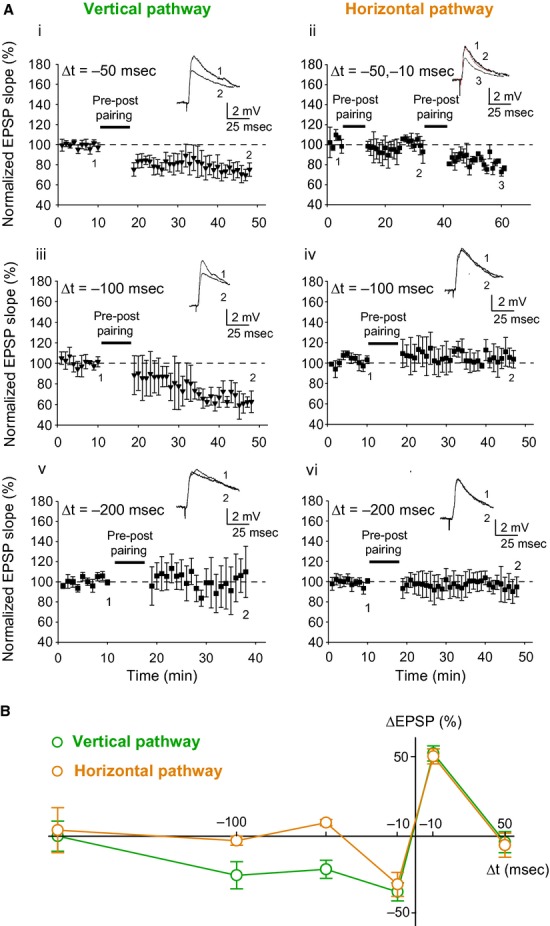
Time window for STDP at vertical and horizontal inputs. (A) Post‐before‐pre pairing protocol induces t‐LTD at vertical but not horizontal inputs for Δt = −50 msec (Ai–ii) and Δt = −100 msec (Aiii–iv), and at neither input for Δt = −200 msec (Av–vi). Δt is the time interval between EPSP onset and peak of postsynaptic spike. Synaptic efficacy was monitored over time in paired pathway (black symbols). Traces show EPSPs from a sample cell before (1) and after (2, 3) post‐before‐pre pairing. (B) Summary plot showing time window for STDP at vertical and horizontal inputs using data from Figs. 1B and 2A. Error bars are SEM. The number of slices for each condition varies between 5 and 8 as indicated in the main text.

A post‐before‐pre pairing protocol with a postsynaptic spike occurring within 50 msec before EPSP onset (Δt = −50 msec) elicited robust t‐LTD at the vertical input (78 ± 6%; *P* < 0.05, one‐sample *t*‐test, *n* = 8; Fig. 2Ai). In contrast, no t‐LTD was observed at the horizontal input with Δt = −50 msec (109 ± 2%; *P* > 0.05, one‐sample *t*‐test, *n* = 6). This was not because the input could not show LTD, as, in the same subset of cells, t‐LTD was subsequently successfully induced with Δt = −10 msec (72 ± 12%; *P* < 0.05, one‐sample *t*‐test, *n* = 6; Fig. 2Aii). Similarly, t‐LTD was seen at the vertical input with Δt = −100 msec (74 ± 9%; *P* < 0.05, one‐sample *t*‐test, *n* = 7; Fig. 2Aiii), but not at the horizontal input (97 ± 3%; *P* > 0.05, one‐sample *t*‐test, *n* = 6; Fig. 2Aiv). A post‐before‐pre pairing protocol with 200 msec delay (Δt = −200 msec), however, did not elicit t‐LTD at either input (vertical, 100 ± 10%; *P* > 0.05, one‐sample *t*‐test, *n* = 6; Fig. 2Av; horizontal, 104 ± 15%; *P* > 0.05, one‐sample *t*‐test, *n* = 5; Fig. 2Avi).

Overall, whereas a narrow time window for t‐LTP induction was seen at both inputs, the time window for t‐LTD induction differed between the inputs, being broader at the vertical than the horizontal input (Fig. 2B), consistent with the broad time window for t‐LTD at vertical inputs reported earlier in rat barrel cortex (Feldman 2000).

### Pathway‐specific induction mechanisms of t‐LTD

**Figure 3.**
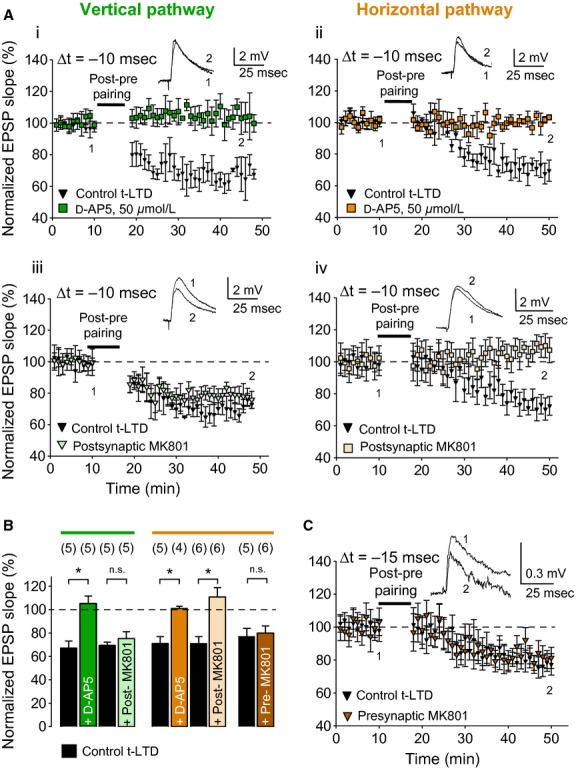
Pre‐ and postsynaptic NMDA receptors in t‐LTD at vertical and horizontal inputs. (A) Bath application of 50 *μ*mol/L d‐AP5 blocked induction of t‐LTD at both vertical (Ai; green squares) and horizontal inputs (Aii; orange squares). In contrast, postsynaptic application of MK‐801 failed to block the induction of t‐LTD at vertical input (Aiii; light green triangles), but blocked t‐LTD at horizontal input (Aiv; light orange squares). *Insets*, EPSP before (1) and 30 min after (2) the pairing protocol. (B) Summary of results. Error bars are SEM. *P < 0.05, unpaired two‐sample *t*‐test. The number of slices used for each protocol is indicated in parentheses at the top of each error bar. (C) Inclusion of MK801 in the presynaptic pipette during paired recordings at horizontal connections failed to block t‐LTD.

To confirm that NMDA receptors are necessary for t‐LTD induction at both vertical and horizontal inputs, the NMDA receptor antagonist d‐2‐amino‐5‐phosphonopentanoic acid (d‐AP5) was used. Bath application of d‐AP5 completely blocked the induction of t‐LTD at both the vertical (105 ± 6%, *P* > 0.05; one‐sample *t*‐test, *n* = 5; Fig. 3Ai and B) and horizontal input (101 ± 2%, *P* > 0.05; one‐sample *t*‐test, *n* = 4; Fig. 3Aii and B).

### t‐LTD at the vertical and horizontal input requires presynaptic and postsynaptic NMDA receptors, respectively

To dissociate the contribution of presynaptic and postsynaptic NMDA receptors in the induction of t‐LTD, the NMDA receptor channel blocker MK801 was included in the presynaptic or postsynaptic recording pipette. As previously reported (Bender et al. 2006; Nevian and Sakmann 2006; Brasier and Feldman 2008; Rodriguez‐Moreno and Paulsen 2008), postsynaptic loading of MK801 failed to block t‐LTD at the vertical input (73 ± 6%, *P* < 0.05; one‐sample *t*‐test, *n* = 5; Fig. 3Aiii and B). In contrast, t‐LTD at the horizontal input was completely blocked when MK801 was included in the postsynaptic recording pipette (111 ± 8%, *P* > 0.05; one‐sample *t*‐test, *n* = 6; Fig. 3Aiv and B). To further confirm the nature of t‐LTD in the horizontal pathway, paired recordings were made from synaptically connected layer 2/3 neurons in the same or a neighboring barrel column. Out of 216 pairs recorded, 13 showed a monosynaptic EPSP, 11 of which were used in plasticity experiments. In five pairs, the t‐LTD protocol induced robust t‐LTD (77 ± 7%, one‐sample *t*‐test, *n* = 5). In contrast to previously reported results using paired recordings at the vertical input (Rodriguez‐Moreno and Paulsen 2008), inclusion of MK801 in the presynaptic recording pipette did not block t‐LTD at the horizontal input (80 ± 6%, *P* < 0.05; one‐sample *t*‐test, *n* = 6; MK801 vs. control, *P* > 0.05; unpaired two‐sample *t*‐test; Fig. 3C), indicating dissociation of presynaptic and postsynaptic NMDA receptor contribution in the induction of t‐LTD at the vertical and horizontal inputs.

### Pathway‐specific locus of expression of t‐LTD

**Figure 4.**
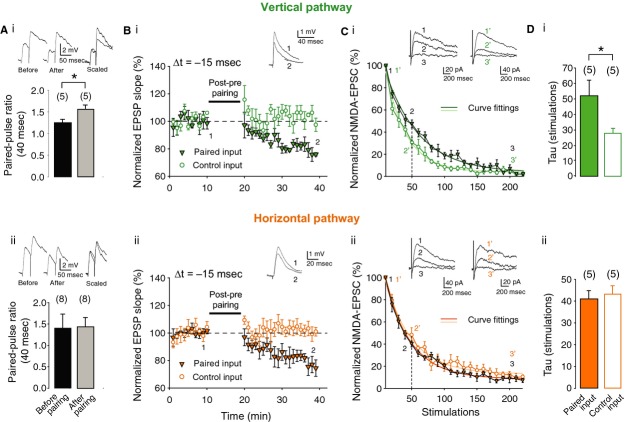
Locus of expression of t‐LTD at vertical and horizontal inputs. (A) Paired‐pulse ratio (PPR) increased after induction of t‐LTD at vertical synapses (Ai) but was unchanged after t‐LTD at horizontal synapses (Aii). *Insets*, representative traces before (left) and after pairing (middle), along with scaled superposed traces (right). (B) t‐LTD at vertical (Bi) and horizontal (Bii) inputs. Traces show representative EPSPs before (1) and after (2) t‐LTD induction. (C) NMDA receptor‐mediated EPSC peak amplitudes monitored over time after bath application of MK801 at the end of the EPSP recordings shown in Bi and Bii. A slower trial‐by‐trial decay of NMDA‐EPSC amplitude was observed in the paired pathway (green triangles) compared with the unpaired pathway (green circles) for the vertical input (Ci), while no such difference was observed for the horizontal input (Cii; orange triangles and circles). The time constant ‘tau’ was calculated for each individual experiment between paired and unpaired control pathway at vertical (Di) and horizontal synapses (Dii). Error bars are SEM. *P < 0.05, paired two‐sample *t*‐test. The number of slices used for each protocol is indicated in parentheses at the top of each error bar.

Since t‐LTD at the vertical input requires presynaptic NMDA receptors whereas t‐LTD at the horizontal input was found to require postsynaptic NMDA receptors, we examined whether the locus of expression might also differ between the vertical and horizontal inputs. To determine the locus of expression after plasticity induction, we measured the paired‐pulse ratio (PPR) and the MK801 blocking rate. Although the PPR increased at the vertical input after induction of t‐LTD (from 1.22 ± 0.08 to 1.56 ± 0.08; *P* < 0.05, paired two‐sample *t*‐test, *n* = 5; Fig. 4Ai), the PPR at the horizontal input did not differ before and after t‐LTD induction (1.41 ± 0.39 vs. 1.43 ± 0.30; *P* > 0.05, paired two‐sample *t*‐test, *n* = 8; Fig. 4Aii), consistent with a presynaptic locus of expression at the vertical input and a postsynaptic locus of expression at the horizontal input. To strengthen this conclusion, we estimated the change in release probability following induction of t‐LTD by analyzing the trial‐by‐trial progressive decrease in NMDA receptor‐mediated currents produced by the use‐dependent NMDA receptor channel blocker MK801. t‐LTD was induced in one pathway at the vertical or horizontal input and compared to an unpaired control pathway (vertical, test 78 ± 5% vs. control 101 ± 5%; *P* < 0.05, paired two‐sample *t*‐test, *n* = 5; Fig. 4Bi; horizontal, test 80 ± 4% vs. control 98 ± 4%; *P* < 0.05, paired two‐sample *t*‐test, *n* = 5; Fig. 4Bii). Twenty minutes after induction of t‐LTD, MK801 was bath applied. Consistent with previous reports (Rodriguez‐Moreno et al. 2011), the vertical paired pathway showed a slower rate of MK801 block than the unpaired pathway (exponential decay time constant, 52 ± 10 vs. 28 ± 3; *P* < 0.05, paired two‐sample *t*‐test, *n* = 5; Fig. 4Ci and Di), indicating a presynaptic change in release probability after t‐LTD induction. In contrast, no difference was observed between the rate of decay of NMDA receptor‐mediated currents in the paired and unpaired pathways at the horizontal input (exponential decay time constant, 41 ± 4 vs. 43 ± 4; *P* > 0.05, paired two‐sample *t*‐test, *n* = 5; Fig. 4Cii and Dii), indicating a postsynaptic locus of expression of t‐LTD at the horizontal input.

## Discussion

This study shows that both vertical and horizontal inputs onto layer 2/3 postsynaptic neurons in mouse barrel cortex display NMDA receptor‐dependent STDP; however, the mechanism underlying the induction and expression of t‐LTD is different in the two pathways. Although t‐LTD in the vertical pathway requires presynaptic NMDA receptors and is expressed presynaptically, t‐LTD in the horizontal pathway requires postsynaptic NMDA receptors for its induction and has a postsynaptic locus of expression. The time window for t‐LTD induction at the vertical input is significantly broader than that at the horizontal input, making STDP depression‐biased at the vertical input (Feldman 2012). These results indicate pathway‐specific differences in the mechanisms of t‐LTD between vertical and horizontal inputs.

Different time windows for induction of t‐LTD at vertical and horizontal inputs suggest that plasticity performs distinct functions at these different synapses. LTD is known to be involved in the refinement of circuits in the developing brain and it might be particularly important for the fine‐tuning of sensory systems. t‐LTD, induced by activation of presynaptic NMDA receptors and expressed presynaptically, may help eliminate synapses during development by reducing release probability at vertical inputs, whereas postsynaptically induced and expressed t‐LTD at horizontal inputs might play a more important role in gain control. Differential NMDA receptor subunit requirement for t‐LTD induction in horizontal (GluN2B) and vertical (GluN2D) pathways has previously been reported in rodent barrel cortex (Banerjee et al. 2009). Presynaptic GluN3A containing NMDA receptor subunits have been shown to be present in juvenile visual cortex gating STDP (Larsen et al. 2011). However, due to the lack of subtype‐selective NMDA receptor antagonists, the exact nature and properties of presynaptic NMDA receptor subunit are yet to be determined at the vertical input onto layer 2/3 neurons in the mouse barrel cortex. Because the time window for t‐LTD at the vertical input is broader than the t‐LTP time window, the vertical input depresses if the temporal relationship between EPSPs and postsynaptic spikes varies randomly (Feldman 2000). This means that spontaneous activity in layer 4, if poorly correlated with postsynaptic spiking, will drive depression at these synapses over time (Feldman 2000). This is different from synapses with more similar time windows for t‐LTD and t‐LTP, as in the tadpole tectum (Zhang et al. 1998) and the hippocampus (Bi and Poo 1998). Functionally, the extended t‐LTD window at vertical inputs might be important for sharpening these connections to ensure that the information transmitted from layer 4 to layer 2/3 represents only the principal whisker of the corresponding barrel. In contrast, the horizontal pathways may process and integrate information from different whiskers and have a greater receptive field (Adesnik and Scanziani 2010).

While it is well established that dendritic location can influence STDP (Froemke et al. 2005; Sjöström and Häusser 2006), input‐specific differences in plasticity are particularly interesting in this circuit because vertical and horizontal inputs spatially overlap across the same regions of the basal dendritic domain of postsynaptic layer 2/3 pyramidal neurons (Lübke et al. 2003; Feldmeyer et al. 2006, Hardingham et al. 2011). Temporally distinct plasticity profiles within spatially overlapping dendritic input domains might be important for the development of circuit mechanisms for the encoding and integration of whisking information. The selective involvement of presynaptic NMDA receptors at the vertical pathway is consistent with the demonstration that NMDA receptor antagonists reduce AMPA‐EPSCs evoked by extracellular stimulation in layer 4, but not layer 2/3 (Brasier and Feldman, 2008). Presynaptic NMDA receptor‐dependent t‐LTD in the vertical pathway is developmentally regulated and disappears by 3–4 weeks of age, both in the mouse barrel cortex (Banerjee et al. 2009) and mouse visual cortex (Corlew et al. 2007), whereas the horizontal pathways have been shown to mature more slowly (Wen and Barth 2011). Therefore, developmentally changing STDP rules along with the development of feedforward and feedback inhibition might play important roles in layer‐specific cortical maturation. In contrast to the conventional STDP rule we report here, which is consistent with earlier reports in mouse visual cortex (Froemke and Dan 2002), synaptic connections between layer 2/3 pyramidal neurons in rat cortical slices were suggested to only show LTD upon coincident low‐frequency presynaptic and postsynaptic activation, independent of the order of the presynaptic and postsynaptic cell firing (Zilberter et al. 2009).

What cellular and molecular mechanisms could be responsible for the distinct t‐LTD window at vertical and horizontal inputs? Although the properties of postsynaptic NMDA receptors appear to be a good candidate for setting the time window for plasticity in the horizontal pathway, the coincidence detector(s) at the vertical pathway are not unambiguously identified. t‐LTD in the vertical pathway is independent of postsynaptic NMDA receptors, and it has been suggested that the coincidence detector instead involves postsynaptic group I metabotropic glutamate receptors, voltage‐sensitive calcium channels and IP3 receptor‐gated calcium stores (Bender et al. 2006). An involvement of presynaptic NMDA receptors as a secondary coincidence detector has not been excluded (Rodriguez‐Moreno and Paulsen 2008; Rodriguez‐Moreno et al. 2010), and signaling via glial cells might also play a role for the temporal requirements of presynaptic and postsynaptic activity at the vertical input (Min and Nevian 2012).

Similar to the induction, the expression of t‐LTD is also precisely regulated. The results of the PPR and MK801 blocking experiments are consistent with earlier PPR measurements (Bender et al. 2006) and fluctuation analysis at the vertical input (Rodriguez‐Moreno and Paulsen 2008). Conversely, no change in presynaptic release probability was observed at the horizontal input, indicating a postsynaptic locus of expression for t‐LTD at this synapse.

In summary, these results demonstrate that similar forms of plasticity on the same postsynaptic neuron may be supported by distinct mechanisms, and suggest that these forms of plasticity may enable these two types of synapses to support different developmental functions in the neocortex.

## Conflict of Interest

None declared.

## Acknowledgments

We thank Olivia Shipton for helpful comments on an earlier version of the manuscript.
